# From lab to field: biological control of the Japanese beetle with entomopathogenic fungi

**DOI:** 10.3389/finsc.2023.1138427

**Published:** 2023-05-10

**Authors:** Tanja Graf, Franziska Scheibler, Pascal A. Niklaus, Giselher Grabenweger

**Affiliations:** ^1^ Extension Arable Crops, Department of Plants and Plant Products, Agroscope, Zurich, Switzerland; ^2^ Department of Evolutionary Biology and Environmental Studies, University of Zurich, Zurich, Switzerland; ^3^ Department of Environmental Systems Science, ETH Zurich, Zurich, Switzerland

**Keywords:** *Popilla japonica*, *Beauveria brongniartii*, *Metarhizium brunneum*, field experiments, virulence, adult, larva, spore injection

## Abstract

The Japanese beetle, *Popillia japonica*, is an invasive scarab and listed as quarantine organism in many countries worldwide. Native to Japan, it has invaded North America, the Azores, and recently mainland Europe. Adults are gregarious and cause agricultural and horticultural losses by feeding on leaves, fruits, and flowers of a wide range of crops and ornamental plants. Larvae feed belowground and damage grassland. To date, no efficient and environmentally friendly control measure is available. Larval populations of other scarab species such as *Phyllopertha horticola* and *Melolontha melolontha* are controlled by applying spores of the entomopathogenic fungi *Metarhizium brunneum* and *Beauveria brongniartii* to larval habitats. Here, we tested this control strategy against Japanese beetle larvae in grasslands, as well as spore spray applications against adults in crops. Using both, large-scale field experiments and inoculation experiments in the laboratory, we assess the efficacy of registered fungal strains against Japanese beetle larvae and adults. *Metarhizium brunneum* BIPESCO 5 established and persisted in the soil of larval habitats and on the leaves of adult’s host plants after application. However, neither larval nor adult population sizes were reduced at the study sites. Laboratory experiments showed that larvae are not susceptible to *M. brunneum* ART 212, *M. brunneum* BIPESCO 5, and *B. brongniartii* BIPESCO 2. In contrast, adults were highly susceptible to all three strains. When blastospores were directly injected into the hemolymph, both adults and larvae showed elevated mortality rates, which suggests that the cuticle plays an important role in determining the difference in susceptibility of the two life stages. In conclusion, we do not see potential in adapting the state-of-the-art control strategy against native scarabs to Japanese beetle larvae. However, adults are susceptible to the tested entomopathogenic fungi in laboratory settings and BIPESCO 5 conidiospores survived for more than three weeks in the field despite UV-radiation and elevated temperatures. Hence, control of adults using fungi of the genera *Beauveria* or *Metarhizium* is more promising than larval control. Further research on efficient application methods and more virulent and locally adapted fungal strains will help to increase efficacy of fungal treatments for the control of *P. japonica*.

## 1 Introduction

The Japanese beetle (*Popillia japonica*) is one of the most important invasive insects threatening the agricultural and horticultural sectors in its invasive range, and is listed as a priority quarantine pest in the European Union (1) and other countries (2, 3). It was accidentally introduced to the USA in the beginning of the 20^th^ century (4) and spread from New Jersey to the west coast, up to Canada, and to the south of the USA (5). Since 2014, the Japanese beetle is present on mainland Europe (6), where it has spread from northern Italy (Piedmont and Lombardy) into southern Switzerland (Ticino), expanding its range each year. Both countries have designated infested zones where phytosanitary measures are in place to limit its spread (7–9).

Unlike many other insect pests, Japanese beetle cause significant damage as both larvae and adults. Larvae are white grubs that feed preferentially on grass roots (4), causing damage to grasslands, football fields, golf courses and other areas with turf (10–12). After emerging in early summer, adults move from the larval habitats to feed on their host plants, where they live for four to six weeks. They are gregarious and polyphagous, feeding on leaves, flowers, and fruits of more than 300 plant species including grapevines, stone fruits, berries, maize, soybean, roses and forest trees (4). Currently, the control of Japanese beetle adults and larvae mainly depends on the use of synthetic insecticides (13, 14), with the cost of damage and control measures estimated at more than $400 million per year in the USA alone (15). Besides these monetary costs, large-scale insecticide applications to control this invasive pest pose a risk to the environment and human health (16–18).

To date, no efficient and environmentally friendly control strategy exists against Japanese beetles (14). However, several biological agents have the potential to control this species, including parasitic nematodes (*Steinernema* sp. and *Heterorhabditis* sp.), bacteria (*Paenibacillus popilliae* and *Bacillus thuringiensis* var. *galleriae*), and entomopathogenic fungi (*Metarhizium* spp. and *Beauveria* spp.; 13, 14, 19–21). Entomopathogenic fungi have proved effective against many insect pests in the Coleoptera, Lepidoptera and Diptera, and fungi of the genera *Beauveria*, *Metarhizium*, *Isaria* and *Lecanicillium* are commercially applied worldwide (22–26). These fungi infect their insect hosts by attaching to and breaching the cuticle (27). For successful infection, the number of adhering spores is crucial, as mortality is dose dependent (28, 29). Once the fungi have reached the hemolymph of the insect, they form blastospores and exploit the nutrients of the insect (30). Upon the death of the host the fungus grows out of the insect to form new conidiospores (31, 32). The duration of fungal incubation can vary from a few days to a couple of months, and depends on the insect species, the virulence of the fungus and environmental conditions (32, 33).

In Switzerland, adults and larvae of native scarab beetles such as the cockchafer (*Melolontha* spp.), the June beetle (*Amphimallon* spp.), and the garden chafer (*Phyllopertha horticola*) are highly susceptible to different strains of *Beauveria brongniartii* or *Metarhizium* sp., depending on the scarab species (33–36). In contrast to the Japanese beetle, these native scarabs almost exclusively cause damage in their larval stages. Thus, the state-of-the-art control strategy against native scarabs consists of culture of the appropriate fungal strain on sterilized barley kernels and application of fungus-colonized barley kernels (FCBK) with a no-till seeder to the larval habitats (meadows, pastures, or turf; 36–38). The Japanese beetle is closely related to these native scarabs and shares a similar ecological niche and life cycle. Under laboratory conditions, several fungal strains (*Metarhizium* spp. and *Beauveria* spp.) have proven successful in infecting and killing Japanese beetle larvae or adults (39–42). Furthermore, Behle et al. (21) achieved a moderate to good control of larvae when applying the fungal strain *Metarhizium brunneum* BIPESCO5/F52 (Bip5) to turf in well-controlled small scale-field experiments. However, there remains a lack of research to test the efficacy of large-scale field application of entomopathogenic fungi against Japanese beetle larvae and adults.

Here, we tested the application of Bip5 against Japanese beetle larvae and adults in the field. We hypothesized that the Japanese beetle can be controlled in a similar way to its native relatives in Europe. Thus, we tested the application of FCBK for control of larvae on the Japanese beetle. Furthermore, we also assessed the efficacy of a spray application of a Bip5 conidiospore suspension to control adult populations. The objective of the field experiments was to monitor the ability of Bip5 to establish and survive in both adult and larval habitats and to measure its effect on insect survival and the damage caused by adult beetles.

Additionally, we carried out two sets of laboratory experiments with the overall aim of assessing the susceptibility of Japanese beetle larvae and adults to three commercially available fungal strains, *M. brunneum* ART 212, *M. brunneum* BIPESCO 5, and *B. brongniartii* BIPESCO 2, under standard laboratory conditions. These experiments revealed host stage-related differences in susceptibility to fungal infection and we hypothesized that they may be explained by cuticular defense mechanisms. We anticipated that larvae have a stronger cuticular defense against soil-borne pathogens than adults, owing to the long subterranean development of the former. Moreover, we expected blastospores to induce faster speed of kill under laboratory conditions due to their fast germination and growth, while more robust conidiospores may be slower in infesting their host.

Our experiments help to assess the potential of entomopathogenic fungi as biocontrol agents against Japanese beetles in the field, and to better understand the mechanisms underlying host-stage-specific virulence of the applied fungal strains.

## 2 Material and methods

### 2.1 Fungal strains

All fungal strains used in the field and laboratory experiments are commercially available. *Beauveria brongniartii* BIPESCO2 (Bip2) was originally isolated by H. Strasser from infected *Melolontha melolontha* (43), *Metarhizium brunneum* BIPESCO5 (Bip5) was isolated in Austria from infected *Cydia pomonella* (44), and ART 212 was isolated from *Agriotes* sp. at Agroscope (Switzerland). To ensure the fitness of the fungal strains, we isolated spores from mycosed cadavers of Japanese beetle (Bip5 and ART 212) and cockchafer (Bip2) larvae from previous inoculation experiments and plated them on selective medium plates (SM: sabouraud 2% glucose agar (SDA) supplemented with cycloheximide (0.05 g/l), streptomycin sulfate (0.6 g/l), tetracycline (0.05 g/l), and dodine (50 mg/l); 45). The F2 generation was grown for two weeks at 22°C and 80% RH in darkness and stored at 5°C after the fungi fully sporulated on the plates.

### 2.2 Meadow field experiments

To test whether the state-of-the-art control of native relatives is effective for Japanese beetles, we carried out two field experiments on meadows infested with Japanese beetle larvae in Piedmont, Italy. Each experiment consisted of 18 plots (9 × 10 m). Experiment 1 (45.6373°N, 8.6087°E, 311 m a.s.l.) was set up in September 2018 when second and third instar larvae were present. Three treatments (six plots each) were established: Bip5 FCBK (applied with a no-till seeder at an equivalent concentration of 10^14^ conidiospores ha^-1^), treatment control (treatment with no-till seeder without FCBK), and a control (untreated). Experiment 2 (45.6354°N, 8.6377°E, 191 m a.s.l.) was set up in May 2019 before adults emerged and laid new eggs. Nine plots received the Bip5 FCBK treatment, the other nine plots were left untreated as control. This second meadow was irrigated once with 30 mm water during peak flight in July 2019 because the soil was very dry.

#### 2.2.1 Fungal inoculum

Bip5 was grown on sterilized barley kernels in polypropylene zipper filter bags (Sac O_2_, Deinze, Belgium; 2 kg unpeeled barley, 1.5 L tap water per bag). The bags were inoculated with sporulating Bip5 culture (F3 generation) that had been grown on complete medium plates (CM: 10 g glucose, 0.36 g KH_2_PO_4_, 1.78 g Na_2_HPO_4_, 1 g KCl, 0.6 g MgSO_4_7H_2_O, 0.6 g NH_4_NO_3_, 5 g yeast extract, 20 g agar per 1 L distilled water; 46; one plate per bag). Bags were incubated for 6–8 weeks at 23°C in the dark and were mixed after approximately two weeks to increase conidiospore production. When fully sporulating, FCBK bags were stored at 5°C until use. Conidiospore concentration was determined by washing the spores from a subsample of each bag with 0.1% aqueous Tween 80 (Sigma-Aldrich, Seelze, Germany) and counting them with a hemocytometer.

#### 2.2.2 Sampling

Soil samples were taken to estimate the number of *Metarhizium* sp. colony forming units (CFU) in the soil before and after the application of treatments. Four soil samples per plot (6 cm diameter × 10 cm depth) were pooled, mixed, and stored in plastic bags at 5°C until processing. The *Metarhizium* sp. CFU g^-1^ of soil were estimated as described by Kessler et al. (37). From each pooled sample, three subsamples of 20–24 g were taken, suspended, and plated on SM. After two weeks of incubation (22°C, 70% RH, darkness), we counted the *Metarhizium* sp. colonies on each plate and determined CFU g^-1^ of soil dry weight. We measured the water content of the soil samples gravimetrically. The mean of the three subsamples per plot was used for statistical analyses.

Larval density in the meadows was estimated by counting all Japanese beetle larvae present in five 20 × 20 × 10–15 cm soil blocks per plot. For statistical analysis, we used the sum of the number of larvae found in the five soil blocks.

### 2.3 Soybean field experiment

To assess whether Bip5 conidiospores can provide control against Japanese beetle adults, we carried out an experiment on an infested soybean field in northern Italy (45.5354°N, 8.6512°E, 177 m a.s.l.). We compared four treatments: 1) Bip5 conidiospore suspension (10^14^ conidiospores ha^-1^ suspended in 600 L ha^-1^ water with 2% Telmion (Omya International AG, Oftringen, Switzerland) as surfactant); 2) a surfactant control (water with 2% Telmion); 3) an untreated control; and 4) a reference application of the insecticide Karate Zeon 1.5 (15 g L^-1^ Lambda-Cyhalothrin, Syngenta, Basel Switzerland, 1.5 L ha^-1^). All treatments were applied with an air-supported trail sprayer. We divided the field into 24 plots (6 plots per treatment) of 21×21 m and assigned the treatments randomly to plots. Treatments were applied at the beginning of the peak flight in early July 2019 in the morning and a second time 6 days later in the evening.

#### 2.3.1 Fungal inoculum

Bip5 conidiospores were produced on barley kernels as described above. Bags with fully sporulating Bip5 were opened and air dried for 2-3 weeks, then conidiospores were removed from the barley kernels (mycoharvester VBS (Agriculture) Ltd., Beaconsfield, United Kingdom), and stored at 5°C. The spore concentration of the powder was determined using a hemocytometer (with the powder in 0.1% aqueous Tween 80 solution). Immediately before application, the conidiospores were formulated with water and 2% Telmion at the field site.

#### 2.3.2 Sampling

To estimate the number of viable *Metarhizium* sp. spores on soybean leaves, we removed nine randomly chosen healthy leaves from all plots except the insecticide treated plots and stored them at 5°C in extraction bags (forming subsamples of three leaves per bag; Bioreba AG, Reinach, Switzerland) until processing. We added 10 mL buffer solution (0.01M PBS, 0.05% (v/v) Tween 20) to each extraction bag and homogenized the leaf samples with the buffer in each extraction bag using a grinding machine. Aliquots of 100 μL of each sample were pipetted and spread on SM plates for incubation (two weeks at 23°C, 80% RH, darkness). We counted *Metarhizium* sp. CFU on each plate, distinguishing them morphologically from other fungi. For statistical analysis, the mean of the three subsamples per plot was used.

To assess the effects on the Japanese beetle population, we counted all adult beetles along 5 m transects of two rows of soybean plants per plot. The sum of all counted beetles per plot was used for statistical analysis. In addition, we scored damage on five randomly selected plants per plot (0: no damage; 1: visible damage but less than half a leaf skeletonized; 2: leaves are green but at least half a leaf is skeletonized; 3: at least one fully skeletonized brown leaf). For each plot, the damage scores of the five plants were summed for statistical analysis.

The effect of the Bip5 conidiospore treatment and the surfactant control on the mortality of beetles was assessed immediately after the first spray application. Ten adults per plot were collected, placed individually in 90 mL plastic tubes filled with moist peat and hazelnut leaves as food, closed with a perforated lid, and incubated for five weeks (23°C, 60% RH, day–night cycle of 16:8 h). We replaced food weekly and checked for mortality at the same time.

### 2.4 Laboratory inoculation experiments

We carried out two sets of laboratory experiments where we combined different application methods (spraying, injection) with different fungal strains (Bip2, Bip5 and ART 212), spore types, and respective control treatments (**Table 1**). All treatments were applied to either Japanese beetle larvae or adults and the timing of the experiments was adjusted to the life cycle of the insect.

**Table 1:** Treatment combinations in the laboratory inoculation experiments.

	Treatment	Spore type	Application method
Experiment 2020			
	Control	None	None
	Tween 0.01%	None	Spray
	Bip2	Conidiospores	Spray
	Bip5	Conidiospores	Spray
	ART 212	Conidiospores	Spray
Experiment 2021			
	Control	None	None
	Tween 0.01%	None	Spray
	H_2_O	None	Injection
	Bip2	Conidiospores	Spray
		Blastospores	Spray
		Blastospores	Injection
	Bip5	Conidiospores	Spray
		Blastospores	Spray
		Blastospores	Injection

Experiments were performed using third instar larvae and adult Japanese beetles (Experiment 2020, adults in June/July, larvae in October; experiment 2021, adults in July, larvae in September). We used five replicates per treatment combination, and 15 individuals per replicate. For the spray applications, we used 10^7^ spores mL^-1^ or 0.01% (v/v) Tween 80. For injections, we used 0.2 μL of a suspension containing 10^6^ spores mL^-1^ (approx. 200 spores per insect) or deionized water.

#### 2.4.1 Insects

Japanese beetle adults and larvae were collected from wild populations in an infested area in the Swiss-Italian border region. Prior to experiments, adults were kept for 2–9 days in a refrigerator (5–6°C, to maintain their fitness) in groups of approximately 50 animals, in plastic containers containing moist peat and vine or hazelnut leaves as food. Larvae were kept for 26–32 days (experiment 2020) and 3–9 days (experiment 2021), individually in six-well cell culture plates filled with moist peat and slices of carrot as food (23°C, 60% RH, day–night cycle of 16:8 h). Before exposure to treatments, larvae were cooled in a refrigerator (5–6°C, for approximately 2 h).

#### 2.4.2 Spore suspensions

F2 generation plates of the fungal strains served as starting material for all spore suspensions. Conidiospore suspensions were prepared by re-plating the F2 generation on SM plates and washing spores off fully sporulating cultures (F3) with 15 mL of sterile Tween 80 solution (0.1% v/v). We removed mycelium and other large particles from the suspensions by vacuum-filtration (Miracloth filter, Merck KGaA, Darmstadt, Germany). Final spore concentration was adjusted to 10^7^ conidiospores mL^-1^ by adding sterile deionized water after counting using a hemocytometer.

Blastospore suspensions were obtained from liquid medium cultures (medium: 3% sucrose, 2.5% yeast extract, 1% peptone and 1% barley flour in 500 mL deionized water) inoculated with six to eight 7 mm-diameter plugs from F2 generation plates. After incubation on an orbital shaker at 250 rpm and 28°C for 3 days for Bip5, and 25°C for 4 days for Bip2, we filtered the liquid cultures through Miracloth to remove the mycelium and washed the remaining blastospores to remove the ingredients of the medium and mycotoxins produced by Bip2 and Bip5 (centrifugation at 1174g for 20 min, removal of supernatant, resuspension in deionized water, second centrifugation, resuspension in deionized water and filtration using Miracloth). We determined the spore concentration using a hemocytometer and adjusted to 10^7^ blastospores mL^-1^ by adding deionized water. For the injection of blastospores, we diluted 1 mL of the blastospore suspension to 10^6^ spores mL^-1^.

#### 2.4.3 Experimental procedure

For the spray treatments, we applied the respective spore solution using a 30 mL spray flask; each insect received one spray dose from each side. For the injection treatments, we used microsyringes (10 μL, G31 injection needle, Hamilton Company, Reno, Nevada) to inject 0.2 μL of the respective suspension behind the third leg of the insect. Germination rates were higher than 95% for all spore solutions (quantified by pipetting three times 50 μL of spore suspension on CM, incubation for 24 h (conidiospores) or 12–18 h (blastospores), and counting 100 spores at 40× magnification).

After inoculation, insects were held individually in insect tubes filled with moist peat and hazelnut leaves (adults) or carrot slices (larvae) as food and closed with a perforated lid. All tubes from one treatment and one replicate were kept together in a plastic box, and these boxes were randomly placed on two racks in a climate-controlled room (23°C, 60% RH, day–night cycle 16:8 h). We assessed the mortality and fungal infection of the insects weekly and replaced food at the same time (adults 4 weeks, larvae 10 weeks). Mycosed cadavers were stored at 5°C and fungal spores were isolated from the cadavers on SM and grown for 2 weeks. The isolates were kept at 5°C until used for genetic analysis.

#### 2.4.4 Genetic analysis

From the experiments in 2021, we selected fungal isolates according to morphology from each treatment to confirm their identity using genetic analysis (simple sequence repeats; SSR). Isolates were spread on CM plates covered with filter paper. After 4–5 days of incubation, the mycelia were scraped off the filters, transferred to 2 mL Eppendorf tubes and frozen at -70°C. The frozen mycelia were lyophilized, and cells were disrupted with glass beads (3 mm and 1 mm) in a FastPrep-24 homogenizer (MP Biomedicals, Eschwege, Germany; 25 s at 6 m s^-1^). We extracted the DNA (sbeadex plant kit and King Fisher Flex Purification system, Thermo Fisher Scientific, Waltham, Massachusetts) and standardized the samples to 5 ng DNA μL^-1^.

We used six SSR markers in two primer pair sets for each species to analyze fungal genotypes (Bb1F4, Bb2A3, Bb2F8, Bb4H9, Bb5F4, Bb8D6 for *B. brongniartii*; 47; Ma2049, Ma2054, Ma2063, Ma2287, Ma327, Ma195 for *M. brunneum*; 48, 49). Reference strains were included for both species (*B. brongniartii*, Bip2 and Bip4; *Metarhizium* spp., Ma714, Ma500 and Bip5). Multiplex PCRs and fragment size analyses were performed as described by Mayerhofer et al. (50) and Fernandez-Bravo et al. (51).

### 2.5 Data analysis

For all field experiments, data collected after treatment at multiple time points were aggregated at plot level (the unit of replication of the applied treatments). We modeled the variables in dependence of the applied treatments by using linear models summarized in analysis of variance (ANOVA) tables. These analyses were performed for all CFU data, data on larval densities in the soil of the meadow field experiments, and the abundance of adult beetles and damage rate of the soybean field experiment. The dependent variables of the two meadow field experiments were square-root transformed before aggregation to increase homoscedasticity. To test for different temporal dynamics in the different treatments, we first regressed the dependent variables against time, and then analyzed the temporal trends (slopes) with a one-sample t-test. This two-step procedure avoids the modeling of serial residual correlation structure, without loss of information. CFU data from both meadow field experiments were square-root-transformed before regression to increase homoscedasticity.

For the meadow field experiment 2 and the soybean field experiment, we expected a natural gradient in the insect populations on the study sites due to their surroundings. Therefore, we added to each of the datasets the variables X and Y to account for the order of the plots. We included those variables as factors into our analysis and fitted them before the applied treatments.

Insect mortality was analyzed using discrete-time hazard models. Specifically, we used a binomial generalized linear model with mortality measured during intervals as dependent variable, with complementary log–log link and the logarithm of the interval length as offset (ASReml-R V4 package, VSNi, Hemel Hempstead, UK). The fixed-effect terms were, in this sequence, interval, the experimental treatments, and the interaction between interval and these treatments. Note that an interaction between interval and any of the treatment indicates a deviation from proportional hazards. The box that harbored the group of initially 15 insects was fitted as random effect. To further inspect effects of the experimental treatments, we decomposed these into a series of individual contrasts and interactions with the time intervals. See results for details.

## 3 Results

### 3.1 Meadow field experiments

**Figure 1:**
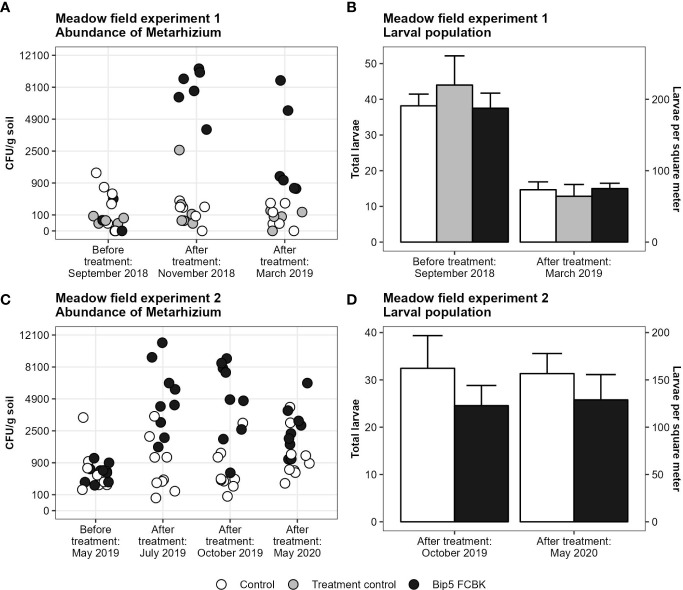
Number of *Metarhizium* CFU g^-1^ soil on square-root transformed y-axis **(A, C)** and mean number of larvae with standard error **(B, D)** before and after treatments.

The application of Bip5 FCBK increased the abundance of *Metarhizium* sp. CFU in the soil in both experiments (**Figures 1A, C**; experiment 1, *F*_1, 15_ = 145, *P* < 0.001; experiment 2, *F*_1, 14_ = 51, *P* < 0.001). Despite increased CFU densities, Bip5 FCBK applications failed to reduce the larval populations significantly (**Figures 1B, D**). In experiment 1, we found no effect of Bip5 FCBK treatment on the larval population (*F*_2, 15_ = 0.5, *P* > 0.5); however, the population decreased over winter by approximately 50% irrespective of the treatment (*t*_17_= 10.2, *P* < 0.001). In experiment 2, the larval population was in general lower in Bip5 FCBK treated plots compared to the control, but the effect was non-significant (*F*_1, 14_ = 3.3, *P* = 0.089). We did not find high winter mortality in experiment 2. *Metarhizium* sp. CFU numbers decreased over winter in Bip5 FCBK treated plots in both experiments (slopes differ from 0, experiment 1, *t*_5_ = -2.6, *P* < 0.05; experiment 2, *t*_8_ = -2.7, *P* < 0.05) but remained constant in the control and treatment control (slopes do not differ from 0, experiment 1, *t*_11_= -1.4, *P* > 0.1; experiment 2, *t_8_* = 0.8, *P* > 0.1).

### 3.2 Soybean field experiment

**Figure 2:**
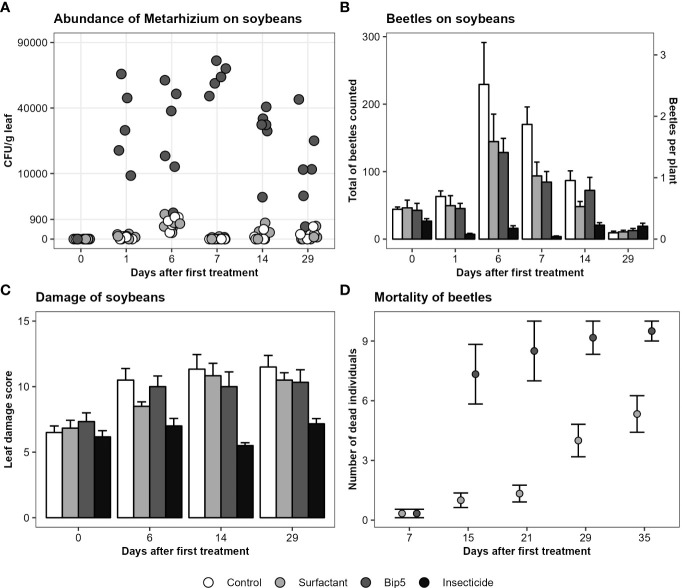
**(A)** *Metarhizium* CFU g^-1^ leaf tissue on the square root transformed y-axis, showing the mean values of the three samples per plot. **(B)** Mean and standard error of total number of beetles counted per plot. **(C)** Mean and standard error of leaf damage score per treatment. **(D)** Mortality of beetles (mean and standard error) collected from the experimental field after the first spray application and incubated in the laboratory. Bip5: Bip5 conidiospore suspension, Control: untreated control, Insecticide: Karate Zeon, Surfactant: surfactant control.

No *Metarhizium* sp. CFU were found on soybean leaves before treatment and the application of Bip5 conidiospores increased the number of *Metarhizium* sp. CFU g^-1^ leaf tissue significantly (**Figure 2A**; **Table 2**). CFU numbers decreased in Bip5 condiospore treated plots over time (slope differs from 0, *t*_5_ = -3.1, *P* < 0.05), but the treatment effect persisted to the last sampling at day 29.

**Table 2:** Analysis of the soybean field experiment.

Dependent variable	Term	df	ddf	*F*	*P*
Log (CFU)					
	Bip5 ↔ Surfactant, Control	1	15	145	**<0.001**
	Control ↔ Surfactant	1	15	0.06	>0.5
Damage					
	X	1	18	1.04	>0.1
	Y	1	18	23.6	**<0.001**
	Insecticide ↔ all other treatments	1	18	29.0	**<0.001**
	Bip5 ↔ Surfactant ↔ Control	2	18	2.30	>0.1
Log (total beetles)					
	X	1	17	1.87	>0.1
	Y	1	17	47.6	**<0.001**
	X × Y	1	17	6.07	**<0.05**
	Insecticide ↔ all other treatments	1	17	156	**<0.001**
	Bip5, Surfactant ↔ Control	1	17	19.3	**<0.001**
	Bip5 ↔ Surfactant	1	17	0.04	>0.5

Bip5, Bip5 conidiospore suspension; Control, untreated control; Insecticide, Karate Zeon; Surfactant, surfactant control. In bold are the *P*-values showing significant differences.

Effect of the different treatments on the dependent variables Metarhizium sp. CFU g^-1^ leaf tissue, damage rating, and the total number of beetles on the soybeans. To disentangle the effects of the different treatments, we applied contrasts. For the analysis of the total number of beetles, we excluded data from day 29, because peak flight ended prior to that date.

The insecticide Karate Zeon significantly reduced the number of Japanese beetle adults on the soybean plants during the peak flight period (**Figure 2B**; **Table 2**). The Bip5 conidiospore suspension and the surfactant control had a moderate effect on beetle abundance on the soybeans, but there was no additive effect of the Bip5 conidiospores above that of the surfactant alone (**Table 2**). The leaf damage score of the insecticide treated plots was significantly lower than for all other treatments, while the Bip5 conidiospore treatment and the surfactant control did not differ from the untreated control (**Table 2**). Damage rates remained at pre-infestation levels when the insecticide Karate Zeon was applied (**Figure 2C**; slopes do not differ from 0, *t*_5_ = 0.9, *P* > 0.1). In contrast, leaf damage increased in all other treatments (slopes differ from 0, Bip5 conidiospore suspension, *t*_5_ = 3.2, *P* < 0.05; surfactant control, *t*_5_ = 7.6, *P* < 0.001; untreated control, *t*_5_ = 4.0, *P* < 0.05). The Bip5 conidiospore application in the field had a strongly significant effect on the mortality of the beetles when incubated in the laboratory, in comparison with the surfactant control (**Figure 2D**; *F*_1, 37_ = 11.5, *P* < 0.01).

### 3.3 Laboratory inoculation experiments

#### 3.3.1 Mortality

**Figure 3:**
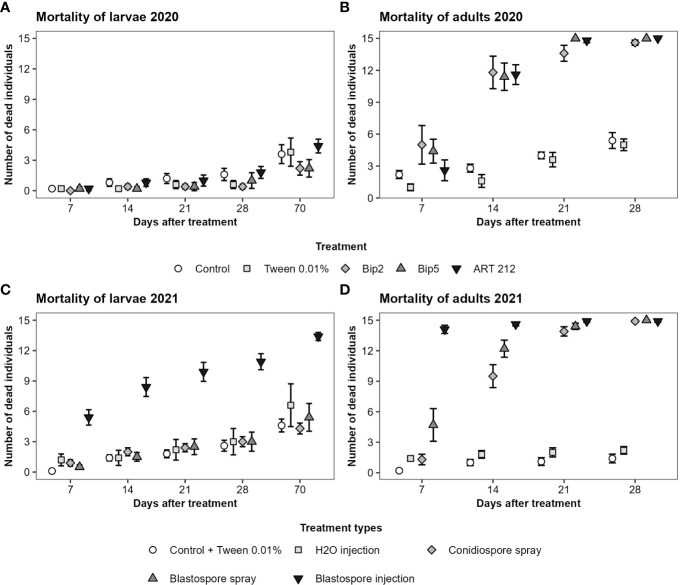
Mortality of Japanese beetle adults and larvae over time. The mortality was assessed over 4 weeks for adults and over 10 weeks for larvae at weekly time intervals. The figures show the mean number of dead individuals and the standard error. For larvae, results of the first 4 weeks and week 10 are displayed. **(A, B)** show the results from the experiments conducted in 2020 with the three fungal strains applied superficially as conidiospores. **(C, D)** show the results from the experiments conducted in 2021. Bip2 and Bip5 were statistically indistinguishable from each other; therefore, we show the aggregated data, broken down by spore type and application method (treatment types).

In general, adult Japanese beetles were more susceptible than larvae to the application of conidio- or blastospores of Bip2, Bip5 and ART 212. The spray application of conidio- or blastospores of all three fungal strains did not affect the mortality of larvae over the 10 weeks of the experiment (**Figures 3A, C**; **Table 3**). In contrast, fungus-treated adults showed elevated mortality already seven days after infection, with a clear effect on day 14 (**Figures 3B, D**; **Table 3**). All mortality effects were due to the applied fungal spores, with the corresponding control treatments having no statistically significant effects (**Table 3**). Neither fungal strains nor spore types differed in the mortality rates provoked in adults or larvae (**Table 3**). Injecting blastospores directly into the insects significantly increased mortality rates of both adults and larvae compared to H_2_O injection (**Figure 3**; **Table 3**). In all experiments, mortality differed between time intervals (**Table 3**). However, we found an interaction between all treatments with blasto-or conidiospores and time intervals only for adults and not for larvae (**Table 3**).

**Table 3:** Analysis of the laboratory inoculation experiments. Effect of the different treatments on the mortality of Japanese beetle adults and larvae.

Term	Larvae				Adults			
Experiment 2020	df	ddf	*F*	*P*	df	ddf	*F*	*P*
**Time**								
Interval	9	178.2	2.88	**<0.01**	3	51.0	17.05	**<0.001**
**Controls**								
Control ↔ Tween 0.01%	1	16.1	0.18	>0.5	1	35.1	0.06	>0.5
Interval × Control ↔ Tween 0.01%	9	178.2	1.38	>0.1	3	49.6	0.84	>0.1
**Fungal treatments**								
Tween 0.01% ↔ Bip2, Bip5, ART 212	1	16.7	0.31	>0.5	1	31.1	45.27	**<0.001**
Bip2 ↔ Bip5 ↔ Art 212	2	15.6	0.55	>0.5	2	15.6	0.55	>0.5
Interval × Control, Tween 0.01% ↔ Bip2, Bip5, ART 212	27	178.2	0.94	>0.5	8	50.8	3.81	**<0.01**
Interval × Bip2 ↔ Bip5 ↔ Art 212	18	178.2	0.39	>0.5	5	51.0	1.76	>0.1
Experiment 2021	df	ddf	*F*	*P*	df	ddf	*F*	*P*
**Time**								
Interval	9	316.7	3.44	**<0.001**	3	83.5	27.03	**<0.001**
**Control treatments**								
Control ↔ H_2_O injection ↔ Tween 0.01%	2	37.6	0.82	>0.1	2	92.6	0.16	>0.5
Interval x Control ↔ H_2_O injection ↔ Tween 0.01%	18	315.7	1.04	>0.1	3	80.1	0.18	>0.5
**Spray treatments**								
Tween 0.01% ↔ Blastospores^1^	2	38.2	0.95	>0.1	2	34.9	30.12	**<0.001**
Time interval × Tween 0.01% ↔ Blastospores^1^	18	315.7	0.83	>0.5	6	81.4	4.24	**<0.001**
Tween 0.01% ↔ Condiospores^1^	2	41.7	0.53	>0.5	2	34.9	27.82	**<0.001**
Interval × Tween 0.01% ↔ Condiospores^1^	18	315.5	0.33	>0.5	6	81.3	3.59	**<0.01**
Blastospores^1^ ↔ Conidiospores^1^	3	40.1	0.56	>0.5	3	23.9	0.08	>0.5
Interval x Blastospores^1^ ↔ Conidiospores^1^	27	315.7	0.40	>0.5	9	82.3	4.10	**<0.001**
**Injection treatments**								
H_2_O ↔ Blastospores^1^	2	26.4	10.13	**<0.001**	2	44.5	45.01	**<0.001**
Interval × H_2_O ↔ Blastospores^1^	18	316.6	0.86	>0.5	5	80.8	0.14	>0.5
**Blastospore application method**								
Injection^1^ ↔ Spray^1^	3	29.6	10.87	**<0.001**	3	28.4	4.36	**<0.05**
Interval × Injection ↔ Spray	27	316.2	0.94	>0.5	8	81.9	6.46	**<0.001**

^1^ These terms include data from Bip2 and Bip5 of the respective application method or spore type. We did not perform separate analyses of the two fungal strains since their effects were statistically indistinguishable. In bold are the *P*-values showing significant differences.

We applied different contrasts to disentangle the effects of application methods, spore types, fungal strains, and time interval.

#### 3.3.2 Mycosis

In the experiment in 2020, *Metarhizium* sp. were observed sporulating on one larval cadaver treated with Bip2 and three cadavers each treated with ART 212 and Bip5. *Beauveria* sp. did not sporulate on any larval cadavers. In contrast, all fungal strains were able to successfully sporulate on the cadavers of adult Japanese beetles. Bip2 (58 mycosed of 73 dead beetles) was slightly more successful in colonizing the treated adults than Bip5 (47 mycosed of 75 dead beetles) and ART 212 (43 mycosed of 75 dead beetles). We found *Beauveria* sp. on two cadavers of adults treated with ART 212. We did not find *Beauveria* sp. sporulating on any cadavers of adults in the control groups, but *Metarhizium* sp. were found on five adults treated with Tween 0.01%.

In the experiment in 2021, all control treatments together contained only three mycosed cadavers (one larva and two adults). One of those isolates was genetically identical with Bip5, the other two isolates differed genetically from Bip2 and Bip5. A few larval cadavers from the spray treatments (blasto- or conidiospores) showed mycosis of *Beauveria* sp. (10 cadavers) or *Metarhizium* sp. (16 cadavers; **Supplementary Table 1**). In contrast, most adult cadavers from the spray treatments were successfully colonized by the respective fungal strain (111 with *Metarhizium* sp., 133 *Beauveria* sp.; **Supplementary Table 1**). The results were similar for adults and larvae in the blastospore injection treatments: Bip2 and Bip5 grew on most of the cadavers of larvae and adults. SSR analysis revealed that the fungal isolates from the cadavers matched genetically with the applied fungal strains in most cases (**Supplementary Table 1**). We found only two unknown *Metarhizium* sp. strains, one each on an adult and larval cadaver treated with Bip5.

## 4 Discussion

Japanese beetle adults and larvae cause major damage in their invasive range. To efficiently protect valuable crops and grassland, a control strategy targeting both life stages is crucial. We tested the impact on Japanese beetle larvae in infested meadows of an application method using *M. brunneum* Bip5 that is well established for the control of scarab larvae native to Europe. In addition, we targeted adults feeding on a soybean field with Bip5 conidiospore spray applications. We found that Bip5 established and persisted in the soil of larval habitats and on the leaves of soybeans. However, neither larval nor adult population sizes were reduced by these treatments at the study sites. Subsequent laboratory experiments revealed that young third instar larvae were not susceptible to superficially applied blasto- or conidiospores of two *M. brunneum* (Bip5 and ART 212) and one *B. brongniartii* (Bip2) strain. In contrast, all spray applications of blasto- or conidiospores of the same strains increased the mortality of adults. Both life stages were susceptible to Bip5 and Bip2 when blastospores were injected directly into the hemolymph of the insects, which suggests that the cuticle is an important factor in determining the difference in susceptibility. We did not detect any differences in the virulence of the different fungal strains, species or spore types which shows the robustness of our findings. Our results suggest that larvae are in general resistant to the three commercially available fungal strains tested here. Thus, we do not see great potential in attempting to adapt to Japanese beetle larvae the state-of-the-art control of native scarabs. However, adults are susceptible to all three fungal strains. Hence, the control of adults with entomopathogenic fungi appears to be more promising.

In our meadow field experiments, we found evidence for good establishment and persistence of Bip5 in the soil, but only a marginal effect of Bip5 FCBK treatment on the abundance of *P. japonica* larvae. This is in contrast with results of Ramoutar et al. (52) and Behle et al. (21), who found clear, albeit variable, control effects when applying Bip5 to turf in well-controlled small-scale field trials. In addition, FCBK application at even lower doses has proven effective in the control of native grubs, and the method is well established (34). Two main factors are probably crucial for the success of the studies cited above. First, clear control effects on white grubs of either Japanese beetle, cockchafer (*Melolontha* spp.), June beetle (*Amphimallon* spp.) or garden chafer (*P. horticola*) were usually obtained when experiments were conducted in moist environments. Additionally, high soil temperature in summer promotes fungal growth and larval infection (53). Furthermore, an intermediate level of moisture and high temperatures have been shown to be most favorable for the infection of *P. japonica* larvae by *Metarhizium anisopliae* (41), while infectiveness is reduced under dry conditions (54). Second, the effectiveness of the fungal treatments is affected by the larval stage targeted (55) with greatest success reported for measures targeting first or second instar larvae (21). We conclude that the optimal setting for the control of *P. japonica* larvae with entomopathogenic fungi of the genera *Metarhizium* are moist soils during summer months, when early larval instars are present, and larvae are actively feeding directly below the sod.

These conditions were not met in our studies. In our first field experiment, Bip5 FCBK were applied in autumn and the soil was moist during the winter season due to frequent rainfall. However, most larvae were in their third instar and the activity of both *P. japonica* larvae and Bip5, was limited by low soil temperatures. In our second field experiment, we applied Bip5 FCBK in May to target eggs and young larval instars. The experimental site received very limited summer precipitation, and the resulting dry soils diminished the efficacy of Bip5. We therefore argue that in the actual infested zone in continental Europe, optimal conditions for entomopathogenic fungi to infect *P. japonica* larvae are rarely met, since moist soils, high temperatures and susceptible larval stages do not coincide.

The control of Japanese beetle adults in the infested zone in northern Italy relies upon insecticide spraying, leaving organic farmers without effective control measures against the invasive pest (phytosanitary service Piedmont, pers. comm.). The spraying of entomopathogenic fungi would offer an environmentally friendly option for organic farmers to protect their crops. Our study shows that Bip5 conidiospores can be applied effectively to soybean plants and that infective propagules persist on leaves over the entire flight period of the Japanese beetle, despite exposure to high temperatures and strong solar radiation. However, Bip5 conidiospore treatment was not more effective than the spore-free surfactant control. The effectiveness of these two treatments averaged around 20–30% (compared to around 70% for the Karate Zeon insecticide treatment; effectiveness calculated according to Abbott; 56), and likely is due to the surfactant in the formulation (57). The lack of an effect of Bip5 conidiospore suspension on the abundance of beetles in the plots was in stark contrast to the difference in the mortality of beetles collected from Bip5 conidiospore treated and surfactant control plots (**Figure 2D**). One explanation may be that a stronger or more rapid effect of the surfactant masked the effect Bip5. The relatively slow action of Bip5 also comes as a disadvantage in comparison to the insecticide Karate Zeon, which caused an immediate knock down of the beetles, with effects that lasted throughout the entire flight period. To accelerate the speed of kill of Bip5 conidiospore treatments, more spores need to come in contact with the beetles, since time-to-death is directly correlated with spore dose (unpublished data; 28). One solution might therefore be to increase the spore concentration of the applied suspension, or to spray repeatedly at shorter time intervals.

Soybean infestation levels in our experimental field were generally low, with an average of less than three beetles per plant. Leaf damage did not exceed one fully skeletonized leaf per plant, even in control plots. This damage level would not have justified the application of an insecticide against Japanese beetles (14), and it may be that effects of the Bip5 conidiospore treatment would have been more readily detectable under a higher insect infestation level.

Our laboratory experiments showed that spray applications of blasto- or conidiospores caused high mortality in adults but not in larvae, independent of the fungal strain. This contrasts previous studies (40, 41, 58); for example, Giroux et al. (40) found no difference in mortality between adults and larvae of Japanese beetle. However, that work used extremely high spore doses (40), which were 8000 times higher than those used here (10^7^ spores mL^-1^, which is commonly used in bioassays). In other laboratory studies, mycelial particles were added to soil as source of infection, resulting in elevated mortality of larvae caused by the applied fungi (41, 58), which contrasts with our findings. To the best of our knowledge, our study is thus the first that reports striking differences in the susceptibility of Japanese beetle adults and larvae to *M. brunneum* and *B. brongniartii*. There is, however, evidence from studies on other insect species that susceptibility to fungal infection differs between developmental stages, especially when larvae and adults do not share the same habitat and are consequently not equally exposed to entomopathogenic fungi (59, 60). Based on these findings, we hypothesize that developmental stages with long periods of exposure to soil-born fungal pathogens show higher resistance to infection than stages that are exposed for only a short time. In Japanese beetles, adults are exposed to attack by soil-borne pathogens during only a very short time at emergence, and in females during oviposition. In contrast, larval stages are exposed for more than half a year, between egg hatch and pupation. It follows that defense mechanisms in the latter should be stronger than in short-lived adults with limited exposure to soil-borne pathogens.

Results from our injection experiments provide evidence that this may be true at least for cuticular defense mechanisms in *P. japonica*. When we injected blastospores of Bip5 and Bip2 directly into the insects to override cuticular defense, we were able to infect both adults and larvae. This indicates that larvae possess a cuticle that protects them efficiently against fungal attack, while cuticular defense mechanisms appear to be negligible in adults. This may seem unexpected, since the heavily sclerotized cuticle of adult beetles appears very robust when compared to the soft-bodied larvae. Several studies have shown that *M. anisopliae* and other entomopathogenic fungi have difficulties penetrating thick and highly sclerotized areas of the integument of other beetle species and cicadas (22, 59, 61). However, spores of *M. anisopliae* preferably attach to the intersegmental membranes and around the setae of locusts (62), and *Beauveria bassiana* presumably penetrates *Tribolium castaneum* larvae through intersegmental membranes (59). Based on these findings, we hypothesize that the intersegmental membranes of adult Japanese beetles may be more prone to fungal attack than the less sclerotized but generally more robust cuticle of their larvae.

However, our results (**Figure 3**) suggest that cuticular defense mechanisms only partially explain the difference in susceptibility of adults and larvae. Larvae were still more resistant to Bip5 and Bip2 than adults, even when blastospores were injected. Adults reached more than 90% mortality after 7 days. In contrast, only around 30% of the larvae were dead after this period, and mortality averaged around 90% after 10 weeks only. This suggests that larvae are not only better protected by their cuticle but also possess more efficient internal defense mechanisms than adults.

Overall, we were able to successfully establish high numbers of *Metarhizium* sp. CFU in the soil by applying Bip5 FCBK. However, these did not effectively control Japanese beetle larvae in the field. Our results were similar in two independent field sites at two different dates, thus encompassing potential differences in susceptibility of *P. japonica* according to the larval stage. We are therefore confident that this finding is robust. We thus cannot recommend Bip5 FCBK application against Japanese beetle larvae in the infested zone in northern Italy as it is used against its native relatives in Europe. While there clearly is room for improvement by adopting more virulent and better-adapted fungal strains (63), and by optimizing their field application, our laboratory experiments indicate that Japanese beetle larvae are generally resistant to entomopathogenic fungi. This conclusion is in line with a literature review of laboratory and field experiments that found that the control of Japanese beetle larvae with entomopathogenic fungi is erratic and thus not recommended (13). Other environmentally friendly alternatives such as the application of entomopathogenic nematodes may be more promising for the control of *P. japonica* larvae (20).

In contrast, the control of adult Japanese beetles with entomopathogenic fungi appears more promising. We found high susceptibility of adult beetles in laboratory settings to Bip2, Bip5 and ART 212. Furthermore, we found that Bip5 can be used in foliar sprays on crops with conidiospores persisting under high UV radiation and heat. Although we did not find clear effects of the Bip5 conidiospore suspension on Japanese beetle abundance or crop damage in a soybean field, we were able to prove Bip5 infections in field-collected adults after application. More efficient spraying techniques or alternative spore dissemination strategies, such as attract and infest approaches (64, 65), may lead to a greater impact on adult *P. japonica* populations. In field experiments in which *M. anisopliae* treated and untreated adult Japanese beetles were released, a significantly lower number of treated individuals were recaptured compared to the control group (66). Furthermore, adults that are attracted to traps equipped with *Metarhizium* spp. conidiospores as inoculum are contaminated with a sufficient spore dose to cause and increase mortality (64, 65). Those contaminated beetles spread the spores to non-infected conspecifics (64, 65), further increasing fungal disease within the population. We suggest that it would be valuable to further test these attract and infest methods in regions with low Japanese beetle population densities to assess whether it may serve as a tool to reduce the flying population and, consequently, further spread of this invasive pest. We conclude that the application of entomopathogenic fungi can be an important tool in an integrated pest management strategy targeted against *P. japonica* adults but not against larvae.

## Data availability statement

The raw data supporting the conclusions of this article will be made available by the authors, without undue reservation.

## Author contributions

TG: conceptualization, methodology, investigation, statistical analysis and writing original draft. FS: investigation and formal analysis. PN: statistical analysis and writing. GG: funding acquisition, project administration, conceptualization, and writing. All authors contributed to the article and approved the submitted version.
