# Supplementary material for: From lab to field: biological control of the Japanese beetle with entomopathogenic fungi
Source: Front Insect Sci. 2023 May 10;3:1138427. doi: 10.3389/finsc.2023.1138427 (PMC10926434; doi:10.3389/finsc.2023.1138427)
Supplement: Supplementary file 1 [file Table_1.pdf]

Supplementary Material

From lab to field: Biological control of the Japanese beetle with entomopathogenic fungi

Tanja Graf[*], Franziska Scheibler, Pascal A. Niklaus, Giselher Grabenweger

*** Correspondence:** Tanja Graf: tanja.graf@agroscope.admin.ch

1 Supplementary Figures and Tables

Supplementary Table 1 Experiment 2021: Mycosis rate and genetic match of the tested samples with the applied fungal strain. The last column shows the number of isolates identical to the applied fungal strain after SSR testing, and the number of isolates tested. Data for each fungal strain are organized in two categories: spray and injection treatments; all control treatments without fungal spores are summarized in one category. In control treatments, only a few mycosed cadavers were found and all of them were genetically analyzed. For the other categories, we chose a subset of isolates depending on morphology and stochastics for genetic analysis.

**Treatment**		**Mortality** dead/total	**Mycosed cadavers**		**Genetic match per samples tested**
			***Beauveria* sp.**	***Metarhizium* sp.**	
**Control treatments**	Adults	25/225	0	2	1 out of 2 Bip5
	Larvae	79/225	0	1	0 out of 1 Bip5
**Bip5 spray treatments**	Adults	150/150	0	110	11 out of 12 Bip5
	Larvae	50/150	0	15	7 out of 8 Bip5
**Bip5 spore injection**	Adults	74/75	0	71	5 out of 5 Bip5
	Larvae	65/75	0	56	5 out of 5 Bip5
**Bip2 spray treatments**	Adults	149/150	133	1	1 out of 1 Bip5<br>17 out of 17 Bip2
	Larvae	47/150	10	1	0 out of 1 Bip5<br>7 out of 7 Bip2
**Bip2 spore injection**	Adults	75/75	73	0	5 out of 5 Bip2
	Larvae	69/75	51	0	6 out of 6 Bip2
